# Adalimumab in Vogt-Koyanagi-Harada Disease Refractory to Conventional Therapy

**DOI:** 10.3389/fmed.2021.799427

**Published:** 2022-01-12

**Authors:** Shizhao Yang, Tianyu Tao, Zhaohao Huang, Xiuxing Liu, He Li, Lihui Xie, Feng Wen, Wei Chi, Wenru Su

**Affiliations:** ^1^State Key Laboratory of Ophthalmology, Zhongshan Ophthalmic Center, Sun Yat-sen University, Guangzhou, China; ^2^Guangdong Provincial Clinical Research Center for Ocular Diseases, Guangzhou, China

**Keywords:** adalimumab, TNF-α inhibitor, Vogt-Koyanagi-Harada (VKH), refractory, treatment

## Abstract

**Background:** No study explores the effectiveness of adalimumab in sight-threatening Vogt-Koyanagi-Harada (VKH) patients in China.

**Objective:** To evaluate the short-term effectiveness and safety of adalimumab (ADA) in patients with sight-threatening Vogt-Koyanagi-Harada (VKH) disease refractory to conventional therapy.

**Methods:** Medical records of VKH patients who had been treated with systemic glucocorticoids and immunosuppressants but whose condition was poorly controlled were collected and analyzed. Primary outcomes comprised of best-corrected visual acuity (BCVA), intraocular inflammation, relapses, and glucocorticoid-sparing effects. Other outcomes included central macular thickness (CMT), intraocular manifestations and adverse events (AEs).

**Results:** Nine refractory VKH patients with a median age of 30 (16, 43) years old were enrolled in this study and received treatment for a median of 10 (7, 11) months. Mean BCVA improved from LogMar 0.63 ± 0.50 (20/72 or 0.36 ± 0.26 in Snellen chart) at baseline to LogMar 0.50 ± 0.37 (20/82 or 0.41 ± 0.28 in Snellen chart) at final visit (*P* = 0.090). The anterior chamber cell grade decreased from 2 (1.75, 3)+ at baseline to 0.5 (0, 1.25)+ cell at final visit (*P* < 0.001). The vitritis grade decreased from 1 (1, 1) + cell at baseline to 0 (0, 1)+ cell at final visit (*P* < 0.001). Patients suffered a median of 1 (0, 2) relapse during treatment. CMT remained stable from 238.50 ± 144.94 μm at baseline to 219.28 ± 77.20 μm at final visit (*P* = 0.553). The mean prednisone dosage decreased from 21.91 ± 18.39 mg/d to 2.73 ± 4.10 mg/d (*P* = 0.005). No severe AEs were found during treatment.

**Conclusions:** The outcomes indicated that ADA was an effective and safe option for VKH patients refractory to conventional therapy by controlling inflammation, preserving visual function and reducing the daily glucocorticoid dose.

## Introduction

Vogt-Koyanagi-Harada (VKH) disease is an autoimmune disorder characterized by severe bilateral granulomatous panuveitis, frequently in association with various systemic manifestations including pleocytosis in the cerebrospinal fluid, tinnitus, alopecia, poliosis, and vitiligo (1–4). Although the prevalence of the disease varies among races worldwide, it has been reported that pigmented races, for instance Asians, Latin Americans, and Middle Easterners, are the primary targets.

Both ocular inflammation and complications, including complicated cataract, secondary glaucoma, choroidal neovascularization and subretinal fibrosis, can result in severe vision loss in VKH patients. The present mainstay principle of treatment in VKH relies largely on early control of intraocular inflammation with systemic high-dose glucocorticoids and immunosuppressive agents. However, the prognosis of VKH remains pessimistic, and prolonged high-dose glucocorticoid administration produces a well-known series of steroid-related side effects.

Although the pathology of VKH remains uncertain, studies have revealed that tumor necrosis factor-alpha (TNF-α) plays a significant role in non-infectious uveitis, including VKH, by inducing the expression of chemokines, adhesion molecules and cytokines associated with ocular inflammation (5–8). Therefore, counteracting TNF-α with TNF-α inhibitors is a reliable strategy in the treatment of non-infectious uveitis.

A fully humanized TNF-α inhibitor, adalimumab (ADA, Humira, AbbVie, North Chicago, Illinois), has achieved promising efficacy in several rheumatic diseases. In 2016, the US FDA granted the indication of ADA in the treatment of non-infectious uveitis. However, only a few case reports have introduced the clinical outcomes of ADA in patients with sight-threatening refractory VKH (9–14). The aim of this study was to evaluate the short-term efficacy and safety of ADA in patients with sight-threatening refractory VKH disease.

## Patients and Methods

We initiated this study in VKH patients who had been systemically treated with glucocorticoids and immunosuppressive agents but whose condition was poorly controlled. All patients had been treated for no <3 months. The medical records of VKH patients who were refractory to conventional therapy were collected and analyzed. This study was granted by the Zhongshan Ophthalmic Center Ethics Committee and the registration number was ChiCTR2000030236. The patients or their guardians were thoroughly informed of the potential hazards, and written informed consent was obtained before initiation of treatment.

Inclusion criteria: (1) had been diagnosed of VKH disease according to the Revised Diagnostic Criteria for VKH disease and the Development and Evaluation of Diagnostic Criteria for VKH disease, and met the profiles of fluorescein angiography (FFA), Indocyanine green angiography (ICGA), and optic coherent tomography (OCT) of VKH (15–17); (2) ocular inflammation was uncontrolled, with continuous degradation of visual acuity in the past 3 months or suffered at least two relapses, which could not be alleviated by increasing dose of systemic medical glucocorticoids and at least one immunosuppressive agent; (3) ocular inflammatory activity was present (anterior chamber cell grade or/and vitritis grade ≥ 1+ cells, optic nerve injury, vasculitis, retinitis or choroiditis); (4) patients had negative T-SPOT test results, or, in the event of a positive test, they had no signs of active tuberculosis and received precautionary anti-tuberculosis treatment.

Patients were excluded if: (1) presence of active systemic infections; (2) coexisting of contraindications to ADA, such as malignant diseases or tuberculosis.

### Treatment

ADA treatment was started with an induction dose of 80 mg subcutaneously, followed by 40 mg 1 week later and 40 mg every other week thereafter. Withdrawal of ADA or extension of the injection interval was based on stability of inflammation remission, patients' wishes and other situations (such as inadequate response, intolerance, side effects or treatment costs). Glucocorticoids (given orally or intravenously) and immunosuppressants, such as methotrexate (MTX), cyclosporine A (CsA) or mycophenolate mofetil (MMF), were prescribed depending on the previous medication and baseline severity of disease, and the doses of glucocorticoids and immunosuppressants were adjusted according to changes in diseases (with MTX ≤ 15 mg per week, CsA ≤ 2 mg/d, MMF ≤ 0.5 g/d at baseline). Children received roughly the same level with adult in dosage per weight unit. Local treatments, such as intraocular anti-vascular endothelial growth factor treatment, glucocorticoid droplets, dexamethasone implants, and anti-glaucoma treatment, were provided according to the conditions.

### Patient Monitoring

The patient evaluations comprised a comprehensive physical examination, a thorough ophthalmic examination and laboratory blood tests (including routine bloodwork, liver and renal function tests, urinalysis, blood chemistry, the T-spot test, and other infectious disease tests). The primary outcomes included best corrected visual acuity (BCVA), intraocular inflammation, relapse and glucocorticoid sparing effects. Other outcomes included retinal morphology, intraocular manifestations and adverse events. All parameters were recorded at baseline and at every visit. All surgical information was recorded, especially for procedures that could improve visual function, such as cataract extraction.

#### Best Corrected Visual Acuity

BCVA was evaluated by Snellen chart and converted to LogMar format. In patients with exceptionally poor visual function of “counting fingers/hand motion/light perception,” BCVA was converted to a quantified visual acuity value for statistical convenience (18).

#### Intraocular Inflammation

The recommendation of the Standardization of Uveitis Nomenclature (SUN) Group in 1990 was used to assess anterior chamber inflammation, and the Nussenblatt scale was used to assess the vitritis grade (19, 20). The SUN Group grading scheme for anterior chamber inflammation is based on the number of anterior chamber cells in 1mm by 1mm slit beam field, and the anterior chamber inflammation is divided into five grades (0, 0.5+, 1+, 2+, 3+, 4+). In the Nussenblatt scale, clarity of optic disc, retinal vessels and nerve fiber layer is used to assess the vitritis into four grades (1+ to 4+).

#### Relapse

A relapse was defined as the emergence or exacerbation of anterior chamber cells, vitritis, mutton-fat keratic precipitates or iris nodules.

#### Central Macular Thickness

CMT was defined as the average retinal thickness within a 1-mm-diameter region in the macular fovea by Cirrus HD-OCT (Carl Zeiss, CA, USA).

#### Intraocular Manifestations

These manifestations included new onset of complicated cataracts, serous retinal detachment (SRD), sunset glow fundus, Dalen-Fuchs nodules, iris nodules, mutton-fat KPs, iris neovascularization, pigmentation scatter, iris nodules (including Koeppe nodules and Busacca nodules), iris synechiae and any other associated complications.

#### Glucocorticoid-Sparing Effect

The glucocorticoid-sparing effect was defined as the reduction of glucocorticoid use at the final visit.

#### Adverse Events

Patients were instructed to report any AEs during the treatment so that their regimen could be evaluated and adjusted. AEs were defined as activation of infections, allergic reactions and any other immunogenicity-related events. The severity of AEs was assessed under the guidance of European Medicines Agency. < https://www.ema.europa.eu/en/documents/other/eudravigilance-inclusion/exclusion-criteria-important-medical-events-list_en.pdf>. This guidance includes the potential severe AEs which resulted in death, life-threatened situations, demanded or prolonged hospitalization, persistenting or significant disability or birth defects.

### Statistical Analysis

For continuous parameters, the mean (SD) or median (interquartile range, or IQR) was used to describe the data. For categorical parameters, numbers or percentages were used. Student's *t*-test was used to compare the BCVA and CMT before and after the treatment. The Wilcoxon test was used to compare pretreatment and posttreatment intraocular inflammation. SPSS ver.25.0 software was used for statistical analysis. *P*-value <0.05 was considered as statistically significant.

## Results

Between April 2020 and May 2021, four males and seven females (a total of 11 patients/22 eyes) were enrolled. The median age at presentation was 30 (16, 43), including one of the participants was a pediatric patient aged 6 years. All patients were biologics-naïve and were at the typical chronic recurrent stage of the VKH course according to the revised diagnostic criteria (RDC) for VKH (16), defined by the presence of sunset glow fundus (except in patient no. 6 and no. 7, who had complete iris synechiae and a completely opaque lens such that the fundus could not be distinguished); all of the patients had suffered relapse attacks before the ADA treatment, and most of them had iris nodules or mutton-fat keratic precipitates (KPs). Four patients had complete VKH, three patients had incomplete VKH, and four had probable VKH (Table 1). The median history of disease before ADA initiation was 8 (4, 26) months.

**Table 1:** Demographics and concomitant treatments of 11 refractory VKH patients.

**Patient no**.	**Age/** **gender/** **laterality**	**Category**	**History (months)**	**Treatment period (months)**	**ADA ongoing at final visit/ADA injections^a^**	**Systemic treatment**	**Concomitant IS at final visit**	**Local therapy**
1 (ZQ)	49/M/OU	C	26	11	–/16	Pred/MTX/CsA	MTX	DEX ivr (OS)
2 (CQM)	41/F/OU	IC	4	10	–/12	Pred/MTX/CsA	Pred/MTX/CsA	
3 (CZJ)	45/M/OU	P	114	7	–/14	Pred/MTX/CsA	Pred/MTX/CsA	
4 (HJZ)	21/F/OU	C	8	10	–/16	Pred/MMF	MMF	
5 (HWZ)	43/M/OU	P	9	12	+/22	MTX	MTX/CsA	
6 (HZM)	16/F/OU	IC	50	7	+/15	Pred/MTX/CsA	CsA/MMF	
7 (TYL)	15/F/OU	C	5	9	+/16	Pred/MTX/CsA	Pred/MTX/CsA	DEX sc (OU)
8 (XHX)	6/F/OU	P	5	12	+/22	Pred/MTX/CsA	MTX/MMF	
9 (WXP)	34/F/OU	C	3	7	+/12	MMF	MMF	
8 (YLP)	30/M/OU	IC	4	10	+/21	Pred/MTX/CsA	Pred/MTX/CsA	TA (OU)
9 (ZQY)	21/F/OU	P	10	11	+/25	Pred/MTX/MMF	MTX	

*Category: C, complete VKH; IC, incomplete VKH; P, probable VKH;*

a *Whether ADA treatment was ongoing at the final visit (+, yes; –, no). ADA, adalimumab; IS, immunosuppressants; OU, oculus uterque (both eyes); OS, oculus sinister (left eye); CsA, cyclosporin A; MTX, methotrexate; MMF, mycophenolate mofetil; TA, triamcinolone acetonide periocular injection; DEX ivr, Ozurdex intravitreal implant; DEX sc, subconjunctival injection of dexamethasone*.

Patients received a median of 10 (7, 11) months of treatment. Apart from ADA, the systemic immunosuppressants that the patients were using as of the baseline visit and the final visit are listed in Table 1. For local treatments, all patients initially received Pred Forte droplets four times/day; the frequency was reduced over time, and the formula was eventually replaced with weaker glucocorticoid droplets. In addition, patient no. 1 received a dexamethasone-delivery implant (Ozurdex, Allergan, California) once in the left eye at baseline due to cystoid macular edema; patient no. 7 received a dexamethasone subconjunctival injection at baseline for severe anterior chamber inflammation; and patient no. 10 received a periocular injection of triamcinolone acetonide once at baseline due to vitritis. Three eyes of two patients (right eye in patient no. 1 and both eyes in no. 7) received cataract surgery during the treatment period, and patient no. 1 received an Ozurdex intravitreal implant in left eye at baseline.

Due to the pandemic quarantine protocol, some patients missed a few monthly visits and temporally discontinued treatment. As of the final visit, seven patients continued their original ADA regimens and the ADA injection interval was successfully extended to 3 weeks in three patients (no. 7, no 8, and no. 9); two patients (no. 2 and no. 3) successful withdrew ADA for stability of ocular inflammation and high cost of ADA; while two patients discontinued ADA due to onychomycosis (no. 1) and lack of response (no. 4).

**Figure 1:**
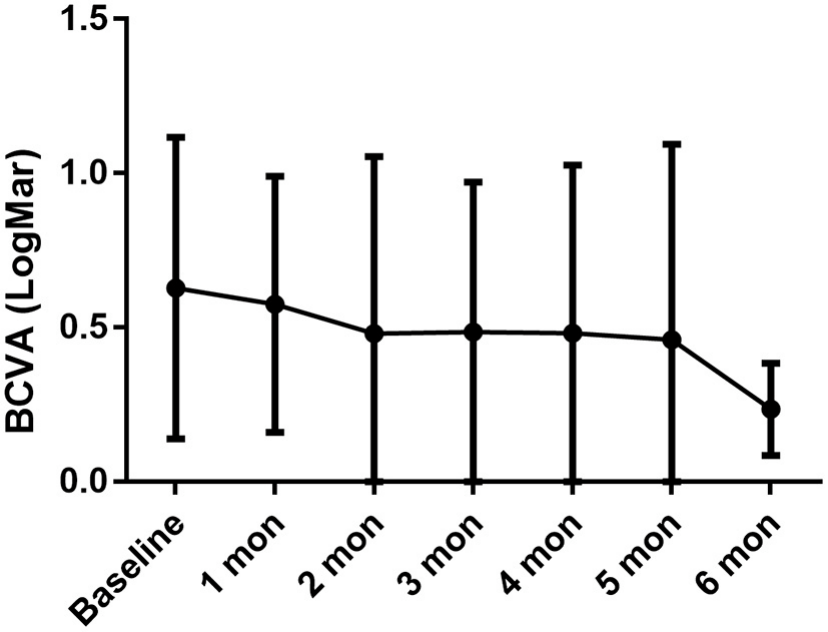
BCVA persistently increased during the treatment period, with baseline LogMar BCVA 0.63 ± 0.49, 0.58 ± 0.41 in 1-month (*P* = 0.033 compared with baseline), 0.48 ± 0.57 in 2-month (*P* = 0.057), 0.49 ± 0.49 in 3-month (*P* = 0.029), 0.48 ± 0.54 in 4-month (*P* = 0.017), 0.46 ± 0.63 in 5-month (*P* = 0.192), 0.24 ± 0.15 in 6-month (*P* = 0.027).

After ~10 months of treatment, the mean BCVA improved from LogMar 0.63 ± 0.50 (20/72 or 0.36 ± 0.26 in Snellen chart) at baseline to LogMar 0.50 ± 0.37 (20/82 or 0.41 ± 0.28 in Snellen chart) at final visit (*P* = 0.090). The monthly changes in BCVA are presented in Figure 1. Patients had a median of 1 (0, 2) relapse during treatment. At baseline, almost all patients had an anterior chamber cell grade of at least 1 (except for patient no. 2, who had a grade of 0.5+ cells in both eyes), and the median grade was 2 (1.75, 3) + cells. After ~10 months of treatment, the anterior chamber cell grade was reduced to 0.5 (0, 1.25) + cell (*P* < 0.001). The vitritis grade was reduced from 1 (1, 1) + cell at baseline to 0 (0, 1) + cell at the final visit (*P* < 0.001). Macular thickness remained stable from 238.50 ± 144.94 μm at baseline to 219.28 ± 77.20 μm at the final visit (*P* = 0.553). The ocular parameters are presented in Table 2. Patients no. 7 and no. 11 had temporary slightly increased intraocular pressure over 21 mmHg, which decreased to normal without medical intervention. Other ocular manifestations that occurred during the treatment and final visit are listed in Table 3.

**Table 2:** Ocular parameters of 11 refractory VKH patients.

**Patient no**.	**Eye**	**BCVA at baseline**	**BCVA at final visit**	**Relapse**	**Anterior inflammation at baseline**	**Anterior inflammation at final visit**	**Vitritis at baseline**	**Vitritis at final visit**	**CMT at baseline (μm)**	**CMT at final visit (μm)**
1 (ZQ)	OD	0.025	0.025	1	1	2	1	0	500	500
	OS	0.25	0.4	1	2	0.5	1	0	724	170
2 (CQM)	OD	0.1	0.2	1	0.5	0	1	1	123	120
	OS	0.1	0.25	1	0.5	0.5	1	0	127	140
3 (CZJ)	OD	0.32	0.32	0	4	0.5	3	0	200	200
	OS	0.25	0.25	0	4	0.5	3	0	200	200
4 (HJZ)	OD	0.4	0.2	1	4	1	1	1	185	205
	OS	0.5	0.2	1	4	1	1	1	185	240
5 (HWZ)	OD	0.63	0.4	2	3	3	1	1	220	220
	OS	0.8	0.25	2	3	3	1	1	220	220
6 (HZM)^a^	OD	0.1	0.2	0	3	0	-	0	-	-
	OS	0.075	0.1	0	3	0	-	0	-	-
7 (TYL)^a^	OD	0.025	0.16	3	2	2	-	0	-	260
	OS	0.025	0.25	3	2	2	-	0	-	260
8 (XHX)	OD	0.5	0.63	2	2	1	2	0	220	220
	OS	0.4	0.63	2	2	1	2	1	220	220
9 (WXP)	OD	0.8	1.0	0	1	0	1	0	240	235
	OS	0.63	0.8	0	1	0	1	0	235	230
10 (YLP)	OD	0.63	0.8	2	2	0	1	0	180	205
	OS	0.63	0.8	2	2	0	1	1	180	210
11 (ZQY)	OD	0.4	0.8	1	3	0	1	0	160	178
	OS	0.4	0.8	1	3	0.5	1	0	174	234

*OD, oculus dexter (right eye); OS, oculus sinister (left eye); BCVA, best corrected visual acuity; CMT, central macular thickness.*

a *Patient no. 6 and no. 7 had complete iris synechiae and a completely opaque lens at baseline, and fundus or OCT examination could not be distinguished. After cataract surgery, fundus and OCT examination in patient no. 7 were feasible*.

**Table 3:** Ocular manifestations during the treatment period.

**Patient no**.	**Baseline ocular manifestations**	**New ocular during the treatment**	**New ocular at final visit**
1 (OD)	Cataract, macular CNV scar	-	IOL
1 (OS)	IOL, CME, KN	-	-
2 (OD)	Pigmentation, subretinal fibrosis, secondary CSC	KN, BN	-
2 (OS)	Pigmentation, subretinal fibrosis, secondary CSC	KN, BN	-
3 (OD)	KN, BN, IOL	-	-
3 (OS)	KN, BN, IOL	-	-
4 (OD)	KN, BN	-	IOP elevation
4 (OS)	KN, BN	-	IOP elevation
5 (OD)	KN, BN	KN, BN	KN, cataract
5 (OS)	KN, BN	KN, BN	KN, cataract
6 (OD)	Iris synechiae, cataract	IOP elevation	-
6 (OS)	Iris synechiae, cataract	IOP elevation	-
7 (OD)	Mutton-fat KPs, BN, Iris synechiae, Sugiura's sign, INV, cataract	KN, BN	IOL
7 (OS)	Mutton-fat KPs, BN, Iris synechiae, Sugiura's sign, INV, cataract	KN, BN, IOP elevation	IOL
8 (OD)	Iris synechiae, cataract	IOP elevation	-
8 (OS)	Iris synechiae, cataract	IOP elevation	-
9 (OD)	-	-	-
9 (OD)	-	-	-
10 (OD)	Cataract, KN, retinal pigmentation	KN, IOP elevation	-
10 (OS)	Cataract, KN, retinal pigmentation	KN, IOP elevation	-
11 (OD)	KN, BN	IOP elevation	IOP elevation
11 (OS)	KN, BN	IOP elevation	-

*OD, oculus dexter (right eye); OS, oculus sinister (left eye); CNV, choroidal neovascularization; IOL, intraocular lens; KN, Koeppe nodules; BN, Busacca nodules; CSC, central serous chorioretinopathy; CME, cystoid macular edema; KPs, keratic precipitates; INV, iris neovascularization; IOP, intraocular pressure*.

**Figure 2:**
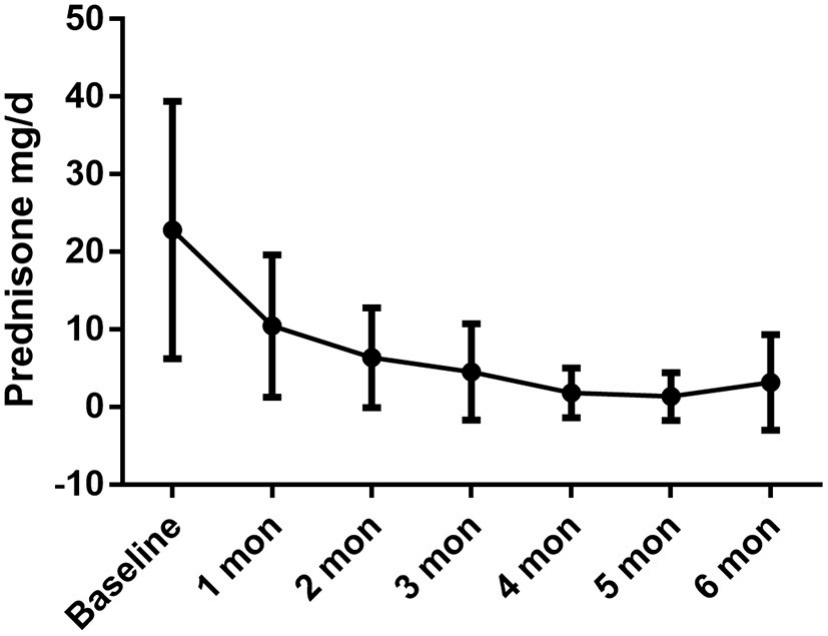
The daily oral prednisone dose was dramatically reduced during the treatment period, with baseline dose of 22.82 ± 16.61 mg/d, 10.45 ± 9.16 mg/d in 1-month (*P* = 0.001 compared with baseline), 6.36 ± 6.43 mg/d in 2-month (*P* = 0.001), 4.55 ± 6.20 mg/d in 3-month (*P* = 0.001), 1.82 ± 3.21 mg/d in 4-month (*P* < 0.001), 1.36 ± 3.08 mg/d in 5-month (*P* < 0.001), 3.00 ± 6.40 mg/d in 6 month (*P* = 0.002).

Regarding the glucocorticoid-sparing effect, the mean prednisone dosage was reduced from 21.91 ± 18.39 mg/d to 2.73 ± 4.10 mg/d (*P* = 0.005). The monthly variations in oral prednisone dosages are illustrated in Figure 2.

**Figure 3:**
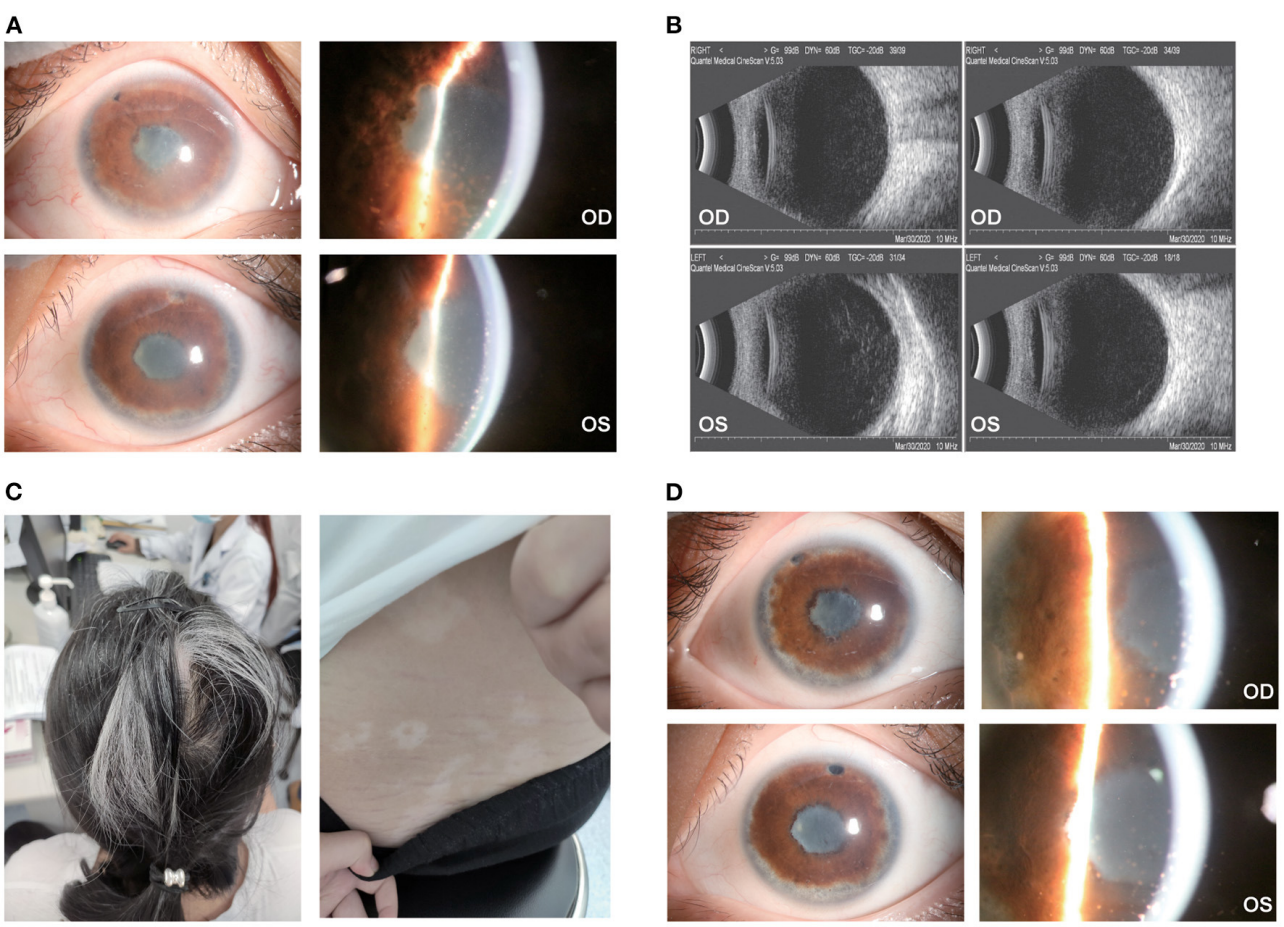
Ocular and extraocular manifestation of patient no. 7. **(A)** The patient had dense and fresh mutton-fat KPs with severe anterior chamber inflammation in the right eye (top) and left eye (bottom); **(B)** dense light spots in the vitreous cavity could be found in both eyes; **(C)** the patient had severe hair loss, white hair and vitiligo along with Tinnitus and headache; **(D)** a month after ADA treatment, the KPs atrophied, and the anterior chamber inflammation relieved in both eyes.

No severe AEs were reported that needed clinical intervention or warranted the discontinuation of ADA treatment. However, mild AEs were found in the following patients, which may or may not be related to ADA treatment: patient no. 1 had onychomycosis; patient no. 4 had a mild urinary infection and delayed ADA for 1 month while receiving antibiotic treatment; patient no. 5 and no. 6 reported an injection-site reaction, which was alleviated by a cold compress; and patient no. 8 had a rash on her back and developed a fever of 37.7°C, both of which disappeared within days; patient no. 10 had occasional fever after long-time work; patient no. 11 had insomnia and occasional premature ventricular contractions which required no medical intervention. A typical case was presented in Figure 3.

## Discussion

VKH accounts for 15.9% of all panuveitis in China and is one of the most common clinical entities in uveitis (21). Previous studies have indicated that treatment of VKH depends largely on early control of intraocular inflammation, and patients treated properly might have an optimistic prognosis for visual function. Unfortunately, many VKH patients do not receive proper treatment, instead receiving delayed or inadequate therapy (suboptimal medication, premature tapering of medication or absence of immunosuppressants), and the disease inevitably progresses to the chronic recurrent stage, in which granulomatous ocular inflammation recurs and most ocular complications emerge, including complicated cataract, secondary glaucoma, choroidal neovascularization, subretinal fibrosis and others (17, 22–24). It has been indicated that ~21% of VKH patients become legally blind (25).

The conventional mainstay of treatment in VKH is combination therapy with systemic glucocorticoids and immunosuppressive agents, including cyclosporine, azathioprine and methotrexate. However, patients with chronic, recurrent, refractory VKH exhibit some resistance and intolerance to conventional therapy (26, 27). In this scenario, remedial treatments are required.

Although the exact etiology remains unknown, TNF-α is regarded as a critical cytokine in the development of uveitis, including VKH. TNF-α inhibitors, which can bind to and deactivate TNF-α, indicate the pathogenesis of uveitis. ADA was the first anti-TNF-α antibody indicated for non-infectious uveitis by the FDA, the European Medicines Agency and the National Medical Products Administration (NMPA) of China. However, there are relatively few focused on the efficacy of ADA in VKH patients. Diaz-Liopis et al. conducted a prospective study on 131 patients with non-infectious uveitis, and ADA was well-tolerated and showed an overall good anti-inflammatory efficacy. However, in that study, very few of the patients had VKH, and the condition of these VKH patients was not described in detail (28). Kwon et al., Su et al. and Jeroudi et al. reported that ADA effectively treated refractory VKH, preserving the patients' BCVA, deactivating ocular inflammation and reducing the daily prednisone dose. However, these studies were case reports with only 1 or 2 patients, and they focused mainly on pediatric patients (9–13). Hitherto, the largest series of VKH patients treated with ADA was a retrospective case series of 14 patients reported by Couto et al. (29). Those investigators concluded that ADA was effective and safe for the treatment of VKH. However, in their study, the degree of vitritis before and after the treatment was unknown, and no ocular manifestations were mentioned. In this study, we explored the efficacy of ADA treatment in refractory VKH patients, and the results indicated that ADA seemed to be a favorable option for these patients. During the treatment period, the ocular inflammation, including anterior chamber inflammation and vitritis, was well controlled in most patients. The relapse frequency of ocular inflammation was relatively small during the treatment. And a substantial cut of the dosage of daily prednisone was achieved without severe side effect.

To our knowledge, this study is the first study on VKH patients treated with ADA in China. In this pilot case series, all patients had received systemic glucocorticoids plus immunosuppressants for over 3 months, but their condition was poorly controlled. We evaluated the short-term efficacy of ADA in refractory VKH patients. ADA seemed to effectively control inflammation and preserve visual function. More importantly, ADA was helpful in reducing the daily glucocorticoid dose without causing additional side effects. Despite local glucocorticoids were applied (including periocular injection of triamcinolone acetonide and Ozurdex intravitreal implant), the pharmacokinetics profile of TA decided that TA did not provide long-term anti-inflammation effect as their half-life periods are merely days or even few hours. However, DEX could provided a relatively long period anti-inflammation effect only for about 3 months (the implant dissolved at 3-month visit). But we only applied Ozurdex implant in the patients with severe ocular inflammation as a reinforcement, and only one patient (1 eye) received Ozurdex implant. Compared with the overall data, we can prudently presume that local therapy had little impact on the treatment of this cohort of patients.

As for the safety profile, no patient in this cohort suffered from severe AEs, which made ADA seemed to be a safe product in management of VKH disease. The most common adverse events in these patients were mild infections (ranging from respiratory system infection, integumentary system and urinary system infection) and mild injection reaction. However, it's necessary to point out that due to the relatively small sample size, the long-term and larger-size observation is still demanded to evaluate the safety of ADA, as some researchers had indicated that ADA might be associated with more severe AEs compared with placebo, including cancers, active or latent tuberculosis, demyelinating disorder and others (30).

However, this study had some unavoidable limitations. First, since this was only a pilot study, the scale of patients was relatively small and it would be worthwhile to carry out additional research exploring long-term efficacy, long-term tolerance and safety. Second, the present study focused mainly on the efficacy of ADA in refractory VKH. ADA is generally considered a second-line treatment in VKH patients. It remains unknown whether ADA could achieve better efficacy in treatment-naïve patients, especially compared to conventional therapy with glucocorticoids and immunosuppressants.

## Conclusion

The outcomes indicated that ADA was an effective and safe option for patients with VKH refractory to conventional therapy; this drug effectively controlled inflammation, preserved visual function and reduced the daily glucocorticoid dose.

## Data Availability Statement

The raw data supporting the conclusions of this article will be made available by the authors, without undue reservation.

## Ethics Statement

The studies involving human participants were reviewed and approved by Zhongshan Ophthalmic Center Ethics Committee. The patients/participants provided their written informed consent to participate in this study. Written informed consent was obtained from the individual(s), and minor(s)' legal guardian/next of kin, for the publication of any potentially identifiable images or data included in this article.

## Author Contributions

WS: conceptualization, writing–review and editing, and supervision. SY, TT, and WS: methodology. SY, TT, ZH, XL, HL, LX, WC, and FW: formal analysis and investigation. SY: writing–original draft preparation. All authors contributed to the article and approved the submitted version.

## Funding

This work was supported by the National Key Research and Development Program of China (2017YFA0105804) and the Local Innovative and Research Teams Project of Guangdong Pearl River Talents Program (2017BT01S138).

## Conflict of Interest

The authors declare that the research was conducted in the absence of any commercial or financial relationships that could be construed as a potential conflict of interest.

## Publisher's Note

All claims expressed in this article are solely those of the authors and do not necessarily represent those of their affiliated organizations, or those of the publisher, the editors and the reviewers. Any product that may be evaluated in this article, or claim that may be made by its manufacturer, is not guaranteed or endorsed by the publisher.
